# Retiming dynamics of harmonically mode-locked laser solitons in a self-driven optomechanical lattice

**DOI:** 10.1038/s41377-024-01736-3

**Published:** 2025-02-02

**Authors:** Xiaocong Wang, Benhai Wang, Wenbin He, Xintong Zhang, Qi Huang, Zhiyuan Huang, Xin Jiang, Meng Pang, Philip. St. J. Russell

**Affiliations:** 1 Department of Optics and Optical Engineering, University of Science and Technology of China, Hefei, 230026 China; 2 Russell Centre for Advanced Lightwave Science, Shanghai Institute of Optics and Fine Mechanics and Hangzhou Institute of Optics and Fine Mechanics, Chinese Academy of Sciences, Shanghai, 201800 China; 3 State Key Laboratory of High Field Laser Physics and CAS Center for Excellence in Ultra-intense Laser Science, Shanghai Institute of Optics and Fine Mechanics CAS, Shanghai, 201800 China; 4 Hangzhou Institute for Advanced Study, University of Chinese Academy of Sciences, Hangzhou, 310024 China

**Keywords:** Solitons, Optics and photonics

## Abstract

Harmonic mode-locking, realized actively or passively, is an effective technique for increasing the repetition rate of ultrafast lasers. It is critically important to understand how a harmonically mode-locked pulse train responds to external perturbations and noise, so as to make sure that it is stable and resistant to noise. Here, in a series of carefully designed experiments, we elucidate the retiming dynamics of laser pulses generated in a soliton fiber laser harmonically mode-locked at GHz frequencies to the acoustic resonance in a photonic crystal fiber (PCF) core. We characterize the self-driven optomechanical lattice, which is distributed along the PCF and provides the structure that supports harmonic mode-locking, using a homodyne setup. We reveal that, after an abrupt perturbation, each soliton in the lattice undergoes damped oscillatory retiming within its trapping potential, while the retiming is strongly coupled to soliton dissipation. In addition, we show, through statistical analysis of the intra-cavity pulse spacing, how the trapping potentials are effective for suppressing timing jitter. The measurements and the theory developed in this work lay the groundwork for studies of the general stability and noise performance of harmonically mode-locked lasers as well as providing valuable insight into generic multi-pulse phenomena in mode-locked lasers.

## Abstract (web summary)

Retiming oscillations of optical solitons in the optomechanical lattice.

## Graphical abstract

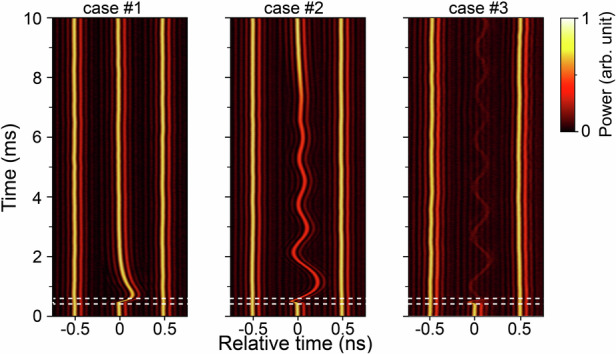

## Introduction

Mode-locked lasers^1^ are of great importance in spectroscopy^2^, frequency metrology^3–5^, micro-machining^6^, optical sampling^7^, biomedicine^8^, and optical information technology^9,10^. As nonlinear dissipative systems, they exhibit a wide range of complex multi-pulse phenomena, for example, harmonic mode-locking^11,12^, which produces a train of regularly spaced pulses at a repetition rate that is a high multiple of the cavity round-trip frequency. Such high-repetition-rate pulse trains are key in many applications^13–15^. Although external modulation has been used to control harmonic mode-locking^16,17^, and intra-cavity interpulse interactions have been studied^18–21^, no comprehensive experimental investigation of the retiming dynamics of multiple pulses in a fiber laser—critical to the stability and noise performance—has been reported to date.

In recent years, few-GHz acoustic resonances in the µm-sized core of a photonic crystal fiber (PCF) have been used to passively and stably mode-lock fiber lasers at a repetition rate that is a high multiple of the round-trip frequency^22–24^. A length of PCF is spliced into a soliton laser cavity^25^, resulting in hundreds of equally spaced solitons in the laser cavity. In the steady state, the GHz-rate pulse train coherently drives the PCF core resonance, which in turn acts back on the soliton pulses, regulating their interpulse spacing and creating a stable optomechanical lattice^26^. The result is a simple and robust platform within which complex soliton dynamics can be studied and their statistical features explored^27,28^.

In this paper, we present the results of a comprehensive experimental and theoretical investigation into the retiming dynamics of solitons in an optomechanically mode-locked soliton fiber laser. Homodyne measurements are used to measure the profile of the optomechanical lattice in the PCF under conditions of stable high harmonic mode-locking. We then introduce abrupt perturbations to selected solitons riding on the lattice using externally launched pulses and investigate the retiming behavior using a phase-space dynamic model. Additionally, we use dispersive time-delay interferometry to measure the interpulse timing jitter and explore how the optomechanical lattice suppresses noise.

## Results

### Formation of optomechanical pulse trapping potential

**Fig. 1:**
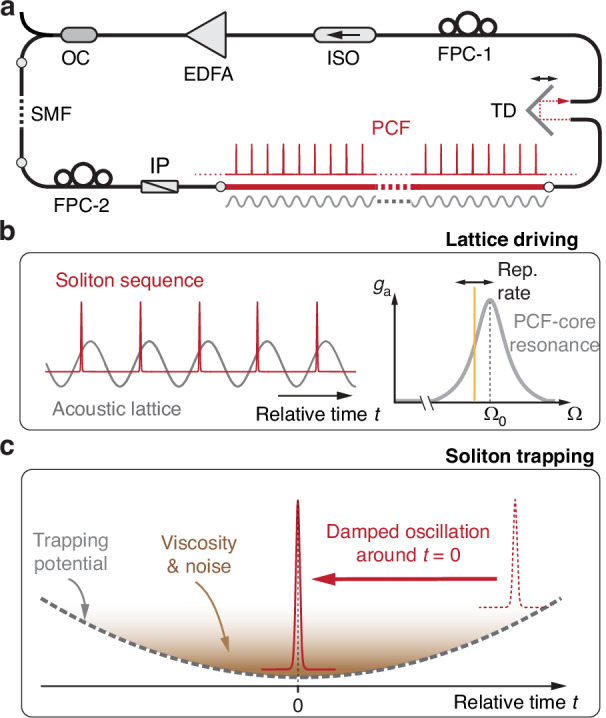
Harmonically mode-locked laser based on optoacoustic interactions in PCF. **a** Sketch of harmonically mode-locked laser cavity based on micro-core PCF and NPR-action. EDFA erbium-doped fiber amplifier, OC optical coupler, SMF single-mode fiber, FPC fiber polarization controller, IP inline polarizer, TD tunable delay line, ISO isolator. **b** The soliton sequence rides on the acoustic field which is coherently driven by the solitons themselves (left) when their repetition rate falls into the acoustic resonance of the PCF core, forming a self-driven optomechanical lattice. The acoustic gain spectrum (right) has a maximal gain (*g*_a_) at the resonance frequency ${\Omega }_{0}$^25^. **c** The soliton in each acoustic cycle is trapped in an effective potential with viscosity and noise and will take damped oscillatory retiming if deviated from the balanced position

A sketch of the high-harmonically mode-locked fiber laser system is shown in Fig. 1a, together with the gain and loss profiles of individual pulses and the optomechanical lattice that regulates the interpulse spacing. An erbium-doped fiber amplifier (EDFA) provides gain and nonlinear polarization rotation (NPR) produces intensity-dependent loss^29^ (see details in “Methods”). The correct balance between gain and loss is critical for initiating mode-locking^30^ and stabilizing the pulse energy, while fiber nonlinearity and anomalous average cavity dispersion result in soliton formation^31^. The soliton sequence coherently drives a PCF core resonance, leading to formation of a sinusoidal acoustic strain wave (the acoustic lattice) traveling at the group velocity of the light^26^ (Fig. 1b). A single soliton rides within each acoustic cycle, trapped by an effective potential that prevents erratic spacing between adjacent pulses (see Fig. 1c). If a soliton is pushed from its stable trapping position, it returns to its original position after oscillating to and fro, damped by gain filtering in the EDFA^32^. This retiming oscillation is inevitably coupled to dissipative effects related to the laser gain and NPR-induced loss. The trapping potential also stabilizes the soliton spacing against noise, which would otherwise cause pulse-timing jitter^33^.

### Measurement of the autonomous optomechanical lattice

#### Homodyne system

**Fig. 2:**
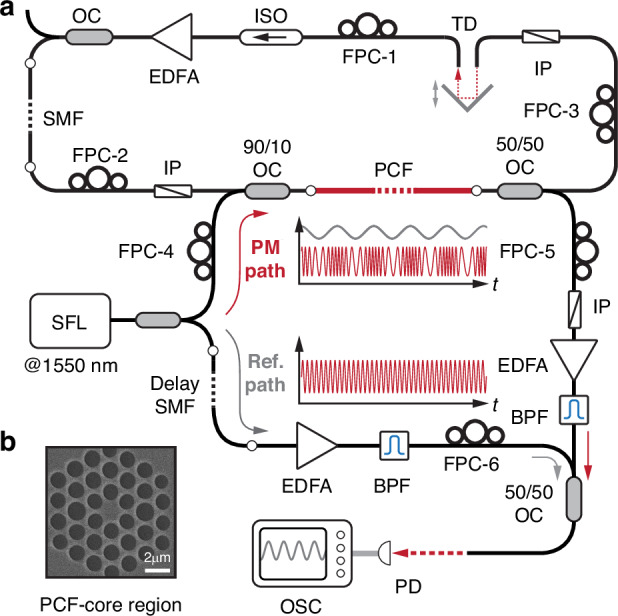
Experimental setup for measuring the optomechanical lattice in PCF. **a** Sketch of the mode-locked laser, incorporating a Mach-Zehnder interferometer for homodyne measurements. SFL single-frequency laser at 1550 nm, BPF band-pass filter, PD photodetector, OSC oscilloscope. **b** Scanning electron micrograph (SEM) of the PCF core structure

In the system investigated (Fig. 2a) the cavity length was 25 m, and the PCF was 0.7 m long and had a core resonance at 1.876 GHz with a FWHM bandwidth of 15 MHz (see Fig. 2b). In the steady state, the laser operated at the 230th harmonic, corresponding to a pulse repetition rate of 1.870 GHz and an acoustic wavelength of ~11 cm along the PCF. To probe the acoustic structure along the PCF (i.e., the autonomous optomechanical lattice driven by the soliton sequence), we incorporated a homodyne system into the laser cavity. The output from a single-frequency 1550 nm laser was split into a reference and a probe signal. The probe was launched into the PCF, and the reference and transmitted probe signals were recombined at a 50/50 coupler working as an interferometer that translates the acoustic phase modulation in the PCF into power modulation that can be recorded by a photodetector and an oscilloscope. To linearize the response, the interferometer was operated at quadrature, and both paths were amplified and adjusted using FPCs to maximize the interference contrast. The probe path in the cavity was terminated by an inline polarizer to avoid perturbing the EDFA.

#### Measurement results

**Fig. 3:**
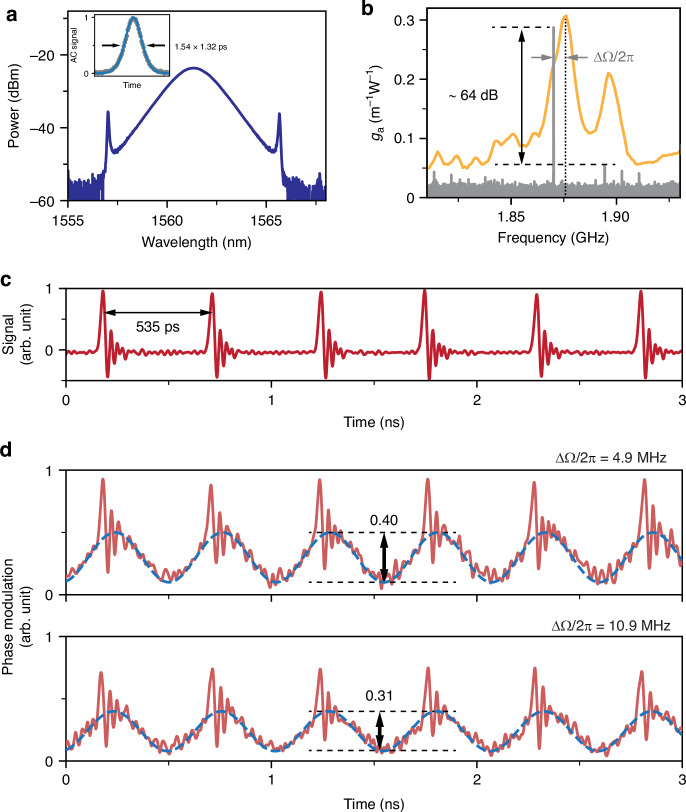
Laser outputs and measured optomechanical lattice in PCF. **a** The optical spectrum and autocorrelation trace (inset) of the output laser solitons. **b** The measured optoacoustic gain in the PCF (orange)^25^ and the power spectrum of the output sequence (gray) with a strong spike at the repetition rate. **c** The output pulse sequence recorded by the oscilloscope. **d** The homodyne power signals normalized to a reference power measured at repetition rate detunings (ΔΩ/2π) of 4.9 MHz and 10.9 MHz

The high harmonic mode-locked laser produced a stable train of pulses at 1562 nm with a fractional bandwidth of 0.0013 and duration 1.3 ps (Fig. 3a). The repetition rate was locked to the core resonant frequency, and the optoacoustic gain had a bandwidth of 15 MHz (Fig. 3b). The repetition rate could be tuned over ~8 MHz using a variable delay line (TD), as explained previously^26^. A typical sequence recorded by the oscilloscope, with pulse spacing 535 ps, is shown in Fig. 3c.

The measured homodyne signal at a repetition rate detuning of 4.9 MHz is sinusoidal in shape, with a single narrow feature (corresponding to a pulse) riding on it in each cycle (Fig. 3d). The signal is the result of elasto-optic and Kerr-related refractive index changes, and clearly confirms that the acoustic wave serves as an optomechanical lattice regulating the interpulse spacing. When the repetition rate was detuned further to 10.9 MHz, the measured amplitude of the acoustic modulation (lower part of Fig. 3d) decreased by ~25%, in good agreement with the predictions of a previous simplified model of the system^26^.

### Oscillatory retiming of laser solitons in the trapping potential

#### Abrupt perturbations using externally launched control pulses

**Fig. 4:**
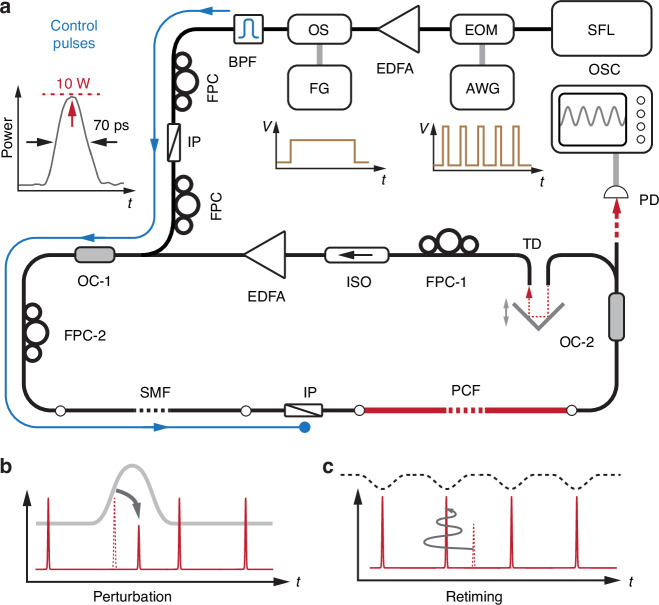
Soliton perturbation induced by externally-launched control pulses. **a** Sketch of the mode-locked cavity including an external system for launching control pulses to perturb individual laser pulses. The blue line marks the path of the control pulses. (Top-left inset: temporal profile of the external pulses.) EOM electro-optical modulator, AWG arbitrary waveform generator, OS optical switch, FG function generator. **b** Temporal shift of a selected soliton in response to the control pulse. **c** Damped retiming oscillation of the soliton in its trap after the perturbation is switched off

To explore the retiming dynamics, we abruptly perturbed a single pulse in the mode-locked sequence and observed its response. Since all the other pulses are undisturbed, the overall structure of the optomechanical lattice is preserved. The perturbation was introduced by externally launched control pulses generated using an electro-optical modulator and spaced by the round-trip time of the laser cavity (Fig. 4a). To avoid disturbing the EDFA, the control pulses were eliminated from the cavity after the SMF using an intra-cavity polarizer (IP).

As the control pulses travel along the 12-m-long SMF, cross-phase modulation causes the selected soliton to move away from its equilibrium position in the trapping potential (see details in Supplement 1). This shift in temporal position is accompanied by changes in group velocity and pulse energy (Fig. 4b). The perturbed pulse train is extracted from the cavity at output port OC-2 (Fig. 4a) and viewed using a fast oscilloscope. When the control pulses are turned off, the perturbed soliton undergoes damped oscillations in relative time, ultimately returning to its equilibrium position^34^ (Fig. 4c).

#### Observations of damped retiming oscillations

**Fig. 5:**
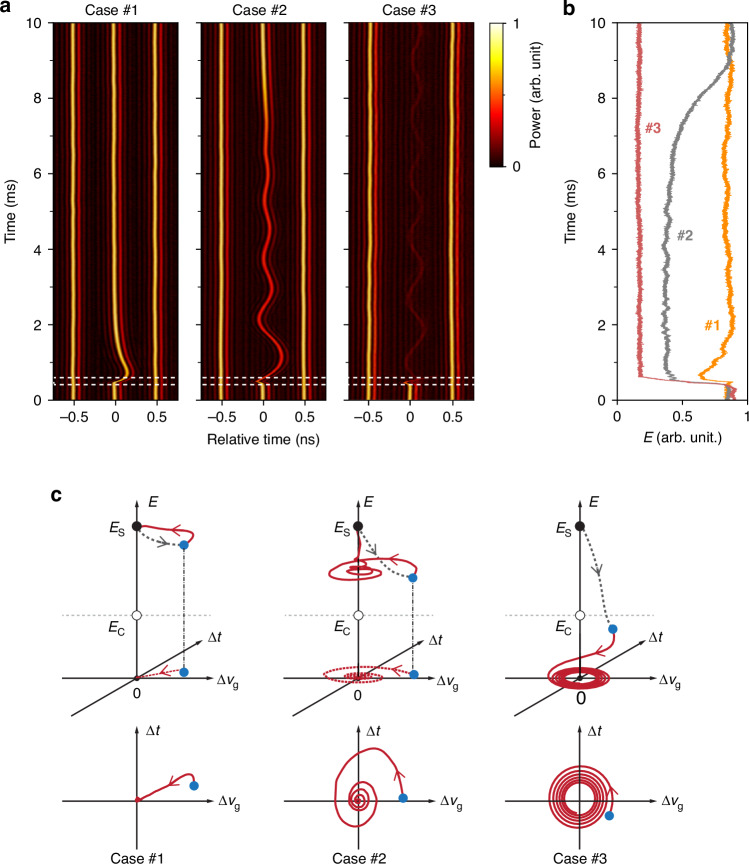
Observation of soliton retiming dynamics after perturbation. **a** Observed retiming dynamics of individual solitons after perturbation by control pulses (the perturbation strength increases from Case #1 to Case #3). The two dashed white lines in each plot indicate the start and end time of the perturbation induced by the control pulses, respectively. Note that in each plot the control pulses only perturb the soliton in the middle, while the two next-neighbor solitons remain unperturbed. **b** Evolution of the soliton energy and **c** phase-space trajectories (red-curves) for the three cases in (**a**) after the end of the perturbation. The dashed gray curves, starting at the black dot and ending at the blue dot, mark the abrupt change in soliton state induced by the perturbation

Three measurements of the retiming dynamics are plotted in Fig. 5a in a time frame moving at the average cavity group velocity. A key result is that the soliton energy falls and the lifetime of the damped retiming oscillations increases as the perturbation strength rises (Fig. 5b). In Case #1, a ~25% decrease in pulse energy is observed, and the 1/e damping time is ~1 ms. In Case #2, the perturbation is stronger, the pulse energy decreases by ~50%, and the damping time is ~6.5 ms, corresponding to ~4 cycles. In both these cases, the pulse energy recovers to its initial value. In Case #3 the perturbation strength is further increased, causing an ~80% decrease in soliton energy. In this case the soliton is no longer able to recover to its initial energy, and intriguingly the retiming oscillations have very low damping, persisting even after the soliton has become very weak, ultimately vanishing into the noise background. In all these cases, the signals from the two unperturbed next-neighbor solitons are included for comparison, confirming that the optomechanical lattice remains stable during the measurements. In addition, the period of the retiming oscillations is determined by the trapping potential in the acoustic lattice and could be varied by adjusting the cavity parameters, in agreement with previous work^26^ (see results in Supplement 1.)

#### Phase-space description and dissipative model

The mechanism underlying this damped oscillatory behavior can be best understood using a phase-space description involving the pulse energy (*E*), and the deviations $\Delta \tau$ in time and $\Delta {v}_{{\rm{g}}}$ in group velocity from the equilibrium values. The measured dynamics (Fig. 5a) can then be mapped on to $E$-$\Delta \tau$-$\Delta {v}_{{\rm{g}}}$ space (Fig. 5c). The unperturbed soliton exists at a fixed-point attractor on the *E*-axis, with $\Delta \tau =0$ and $\Delta {v}_{{\rm{g}}}=0$ (black dots in Fig. 5c). For a small decrease in soliton energy (Case #1), the trajectory is heavily damped and the system moves nearly monotonically toward the attractor from the perturbed point (blue dot). For a stronger perturbation, the soliton energy decreases significantly, and the system follows a spiral trajectory toward the attractor. For a sufficiently strong perturbation, the soliton energy falls below a critical value ${E}_{{\rm{C}}}$ (Case #3), and the system is no longer captured by the attractor, moving instead along a spiral trajectory toward the zero-energy plane, where the pulse disappears.

**Fig. 6:**
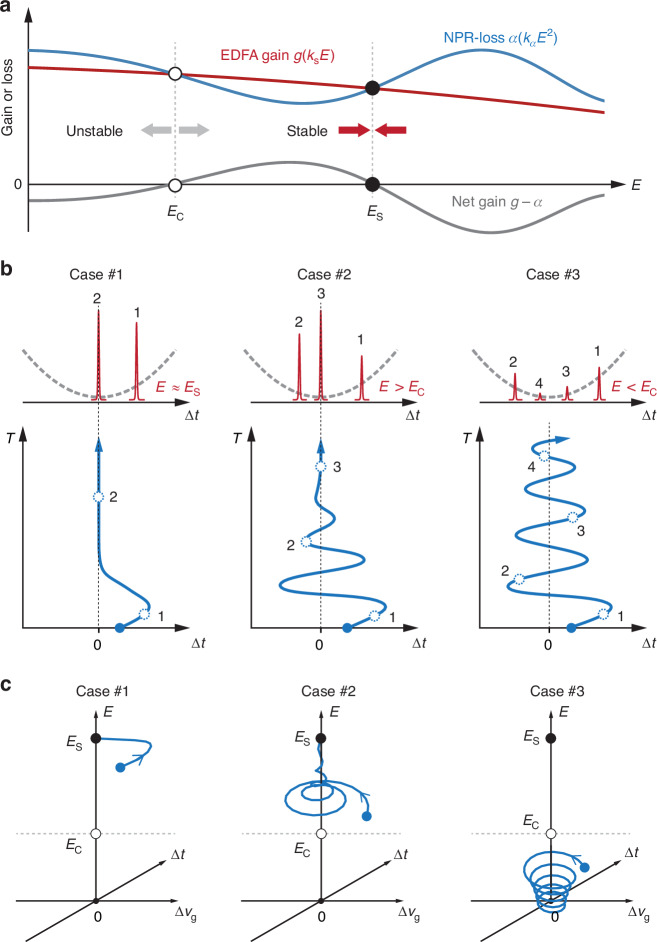
Dynamic model for soliton retiming in a trapping potential. **a** Intensity-dependent NPR-induced loss and energy-saturable EDFA gain leads to a stable pulse energy ${E}_{{\rm{S}}}$, while a critical pulse energy ${E}_{{\rm{C}}}$ exists below which the pulse gradually disappears. **b** Illustration of the key features of the retiming dynamics, for three different initial conditions. **c** The simulated phase-space trajectories based on the dynamic model described by Eqs. (1)–(3) for the retiming processes. Three different initial conditions are chosen corresponding to (**b**)

Following the well-known approach by Haus^1^, we have developed a dynamic analytical model incorporating both retiming^26^ (Fig. 1c) and changes in dissipation caused by laser gain and nonlinear polarization rotation (see Fig. 6a). The pulse energy *E* is governed by:

1 $$\frac{{dE}}{d\tau }=g\left({B}_{{\rm{s}}}\right)E-\alpha \left({P}_{{\rm{s}}}\right)E\approx \left(g\left({k}_{{\rm{s}}}E\right)-{\alpha }_{0}\sin \left({k}_{\alpha }{E}^{2}\right)\right)E$$

where $\tau =T/{T}_{{\rm{R}}}$ is the normalized time, ${T}_{{\rm{R}}}$ the cavity round-trip time, $g\left({B}_{{\rm{s}}}\right)\approx g\left({k}_{{\rm{s}}}E\right)$ the laser gain (which decreases as the soliton bandwidth ${B}_{{\rm{s}}}$ increases (at larger $E$) due to gain filtering^32^), and $\alpha \left({P}_{{\rm{s}}}\right)$ is the loss induced by nonlinear polarization rotation, which depends sinusoidally on the peak pulse power ${P}_{{\rm{s}}}\approx E/{\tau }_{{\rm{p}}}\approx {k}_{\alpha }{E}^{2}$, where ${\tau }_{{\rm{p}}}$ is the pulse duration and ${k}_{\alpha }$ a heuristic constant^29^. As shown in the sketch in Fig. 6a, the combined gain term in Eq. (1) oscillates around zero with increasing *E*. The first zero at $E={E}_{{\rm{C}}}$ has positive slope and is unstable; for $E\, < \,{E}_{{\rm{C}}}$ the pulse energy decays to zero, and for $E \,>\, {E}_{{\rm{C}}}$ it increases until it reaches the second zero at $E={E}_{{\rm{S}}}$, which has a negative slope and is therefore stable; the soliton energy is then clamped at this value. At larger energies, the laser gain falls off so that there are no more zeroes in the combined gain and no further stable points (see details in Supplement 1.)

The temporal shift in pulse position $\Delta \tau$ is directly related to the change in group velocity $\Delta {v}_{{\rm{g}}}$:

2 $$\frac{d\Delta \tau }{d\tau }=-\frac{\Delta {v}_{{\rm{g}}}}{{v}_{{\rm{g}}0}}$$

where ${v}_{{\rm{g}}0}$ is the group velocity at the equilibrium point. Defining the normalized group velocity change as $\Delta {\hat{v}}_{{\rm{g}}}=\Delta {v}_{{\rm{g}}}/{v}_{{\rm{g}}0}$, the time dependence of $\Delta {\hat{v}}_{{\rm{g}}}$ is governed by:

3 $$\frac{d\left(\Delta {\hat{v}}_{{\rm{g}}}\right)}{d\tau }=-\Gamma \left({B}_{{\rm{s}}}\right)\Delta {\hat{v}}_{{\rm{g}}}+F\left(\Delta \tau \right)$$

where $\Gamma \left({B}_{{\rm{s}}}\right)$ is the damping rate, and $F\left(\Delta \tau \right)$ is the “restoring force,” which is linearly proportional to $\Delta \tau$, assuming a quadratic trapping potential.

Equations (2) and (3) can be combined to yield a 2nd-order differential equation:

4 $$\frac{{d}^{2}\Delta \tau }{d{\tau }^{2}}+\Gamma \left({B}_{{\rm{s}}}\right)\frac{d\Delta \tau }{d\tau }-F\left(\Delta \tau \right)=0$$

which describes the behavior of the system when perturbed from its equilibrium position. A key point here is that $\Gamma \left({B}_{{\rm{s}}}\right)$ increases with soliton bandwidth due to gain filtering^32^ so that lower soliton energies lead to narrower pulse bandwidths and slower damping rates^34^ (see details in Supplement 1.)

The retiming dynamics described by this analytical model are summarized in Fig. 6b with three typical cases. In Case #1 the soliton energy after perturbation is only slightly lower than ${E}_{{\rm{S}}}$, and the system response is overdamped, quickly returning to equilibrium. In Case #2 the soliton energy after perturbation is significantly lower than ${E}_{{\rm{S}}}$ but still above ${E}_{{\rm{C}}}$, and the system oscillates with a longer damping time, returning to equilibrium more slowly. In Case #3 post-perturbation soliton energy is lower than ${E}_{{\rm{C}}}$, and thereafter, slowly falls, exhibiting long-lived oscillations that continue until the soliton has almost vanished. The numerical results based on this dynamic model are given in Fig. 6c, showing damped oscillations around ${E}_{{\rm{S}}}$ for $E > {E}_{{\rm{C}}}$ and long-lived oscillations for $E < {E}_{{\rm{C}}}$ that decay slowly toward zero energy, which is in good agreement with the experimental results in Fig. 5.

### Suppression of interpulse jitter in the trapping potential

#### Dispersive time-delayed interferometry

**Fig. 7:**
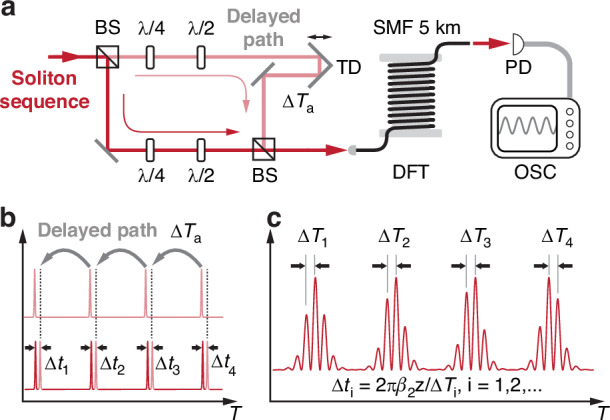
Experimental setup for measuring the interpulse jitter. **a** The DTDI setup for resolving fluctuations in interpulse spacing. BS beam splitter. **b** The pulse train is split into two paths, one of which has a variable delay line. The two paths are then recombined at a beam splitter, and the delay adjusted until there is a train of closely spaced pulse pairs. **c** TS-DFT signal of the pulse-pair sequence, in which the temporal spacing between the fringes is inversely proportional to the temporal spacing of each pulse pair

In the absence of external perturbations, the pulse timing will be influenced by noise sources such as amplified spontaneous emission (ASE)^33^, resulting in unpredictable interpulse jitter. At the same time, the acoustic wave in the PCF, driven by the intra-cavity pulse train, can, in principle, suppress the timing jitter by continuously enforcing a regular pulse spacing. To probe the interpulse jitter, we employed dispersive time-delayed interferometry (DTDI)^35^ to follow real-time fluctuations in interpulse spacing, which are too fast to be resolved by direct detection using fast electronics. In the DTDI setup, the output pulse sequence from the mode-locked laser is divided into two paths, one being temporally delayed before being recombined with the other path (Fig. 7a). The result is a train of closely spaced pulse pairs (Fig. 7b), which can be resolved after time-stretched dispersive Fourier transformation (TS-DFT) over a 5 km length of SMF^36^. The residual pulse spacing is retrieved from the fringe period of the resulting spectral interferogram, with a temporal resolution less than 1 ps^37^ (Fig. 7c). Jitter-induced changes in interpulse spacing can then be monitored by comparing the fringe periods for each pulse pair.

#### Statistical analysis of interpulse jitter

**Fig. 8:**
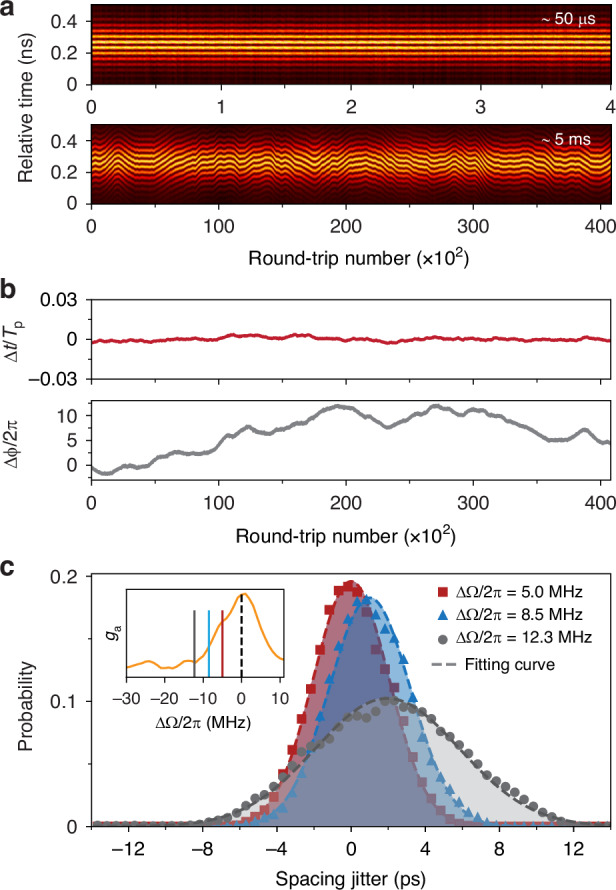
Measured results of the interpulse jitter. **a** The evolution over 5 ms of the TS-DFT signal for an arbitrarily selected pulse pair over consecutive round-trips (zoom-in over 50 μs is also shown). **b** Upper: the retrieved spacing jitter ($\Delta \tau$) from (**a**), relative to the average interpulse spacing ${T}_{{\rm{p}}}$ in the pulse train as it leaves the laser. Lower: the relative phase ($\Delta {{\varphi }}$) between the pulse pair. **c** The probability distribution of the interpulse jitter for three different values of repetition rate detuning. Inset: the optoacoustic gain profile, with the three values of detuning marked

The DTDI signal was recorded using a fast oscilloscope, resulting in 230 real-time sequences of temporal interferograms formed by neighboring solitons. The temporal interferogram of a randomly selected pulse pair is plotted over consecutive round-trips in Fig. 8a. It is clear that the resulting interferograms generally remain stable in the short term (~50 μs) while exhibiting prominent random drifts over longer times (~5 ms). The long-term pulse spacing is plotted in the lower part of Fig. 8b, showing a bounded stochastic oscillation around an average position. The retrieved relative phase between consecutive pulses, in contrast, exhibits an unbounded random-walk (Fig. 8b), indicating that the carrier phase of the solitons are uncorrelated^38^. Analyzing the distribution probability of the retrieved spacings for all 230 pulse pairs over a time scale of 5 ms resulted in a narrowly distributed Gaussian profile centered at the average position, as shown in Fig. 8c. Most importantly, this distribution widens when the repetition rate is detuned from the acoustic resonant frequency, as shown in Fig. 8c, where the full-width-half-maximum jitter distribution broadened from 4.8 ps to 9.2 ps as the frequency detuning was changed from 5 MHz to 12.3 MHz. Note that the center of the distribution shifts slightly due to the change in repetition rate. These results confirm that the optomechanical lattice suppresses timing jitter, with an effectiveness that falls off as the repetition rate is detuned from the acoustic resonance. This is because weaker driving of the acoustic wave (see upper part of Fig. 3d) weakens the trapping potential, reducing suppression of interpulse jitter.

## Discussion

Self-stabilization of a high harmonic mode-locked pulse train can be achieved by strong optoacoustic interactions in a small-core PCF, resulting in the generation of an acoustic wave that, close to cut-off, travels at the group velocity of the light and forms an optomechanical lattice that clamps each laser soliton into a trapping potential. In the presence of external perturbations and intrinsic noise, the optomechanical lattice robustly enforces the pulse repetition rate, stabilizing the interpulse spacing and providing the trapping potentials critical for the suppression of pulse-timing jitter. Our measurements show that the retiming process is strongly correlated with soliton dissipation. We believe that these features will be of critical interest in other dissipative nonlinear systems that support ordered patterns of pulses.

An open question is how the harmonic mode-locking gets started. In the experiments, mode-locking self-initiates from noise without any ordering, whereas the steady state is a highly ordered structure with stable interactions between a regular sequence of solitons and a coherently driven autonomous optomechanical lattice. The retiming dynamics of an individual soliton in the steady state cannot be used to explain how a highly ordered sequence of hundreds of solitons is established^39^. A further interesting question concerns the carrier-wave phase relationship between pulses when the laser is stably mode-locked^38^ (lower part of Fig. 8b). It remains unclear how this is affected by inter-soliton interactions, or whether it can be manipulated through cavity control. The frequency-domain (comb) structure of the laser is also unclear due to the massive number of solitons and their complicated collective behavior. Successive pulses are likely to be uncorrelated in phase, leading to highly stochastic comb structures^38^ that are difficult to resolve using conventional methods, while at the same time offering rich opportunities for manipulating the time- and frequency-domain structures of high-repetition-rate lasers^40–42^.

The reported laser system provides an elegant platform for studying multi-pulse interactions and their dynamics, enriching our understanding of the retiming dynamics of optoacoustically mode-locked fiber lasers, and laying the groundwork for future experimental and theoretical studies of self-stability and noise suppression in generic harmonically mode-locked lasers.

## Materials and methods

### Mode-locked laser

The optomechanically mode-locked soliton fiber laser shown in Fig. 1a of the main text employed a 1-m-long erbium-doped fiber with a peak absorption of 30 dB m^−^^1^ at 1530 nm as the gain fiber. The gain fiber is pumped by two laser diodes at 980 nm with a combined pump power of 1 W. A tunable delay line (TD) is inserted to ensure that one harmonic of the cavity round-trip frequency would fall into the acoustic resonance of the solid-core PCF. Two fiber polarization controllers (FPCs) are used inside the laser cavity for adjusting the polarization bias to enable the self-starting of the laser mode-locking through nonlinear polarization rotation (NPR). A third FPC is inserted between polarizer and the PCF in order to launch the linearly polarized laser light into one principal axis of the PCF. This particular FPC was fixed in all the subsequent experiments, and therefore, we have omitted it in all the setup sketches in the main text (Figs. 1, 2, and 4). By carefully adjusting the FPCs, the laser could be arranged to mode-lock at the 230th harmonic of the cavity round-trip frequency. The output soliton sequence is sent to a diagnostic setup which consists of two ports. At one port, the optical spectrum of the soliton pulses (see Fig. 3a) was recorded directly using an optical spectrum analyzer (OSA, Yokogawa, AQ6374), with a spectral resolution of 0.02 nm. At the other port, the time-domain sequence of the solitons (as shown in Fig. 3c) was recorded using a fast photodetector (PD) and an oscilloscope (OSC, Tektronix, DPO75902SX), with a sampling rate of 50 GSa s^−^^1^ and a detection bandwidth of 15 GHz.

## Supplementary information

Retiming dynamics of harmonically mode-locked laser solitons in a self-driven optomechanical lattice

## Data Availability

The data that support the plots within this paper and other findings of this study are available from the corresponding authors upon reasonable request.

## Code availability

The code used in this paper is available from the corresponding author upon reasonable request.
