# Supplementary material for: Retiming dynamics of harmonically mode-locked laser solitons in a self-driven optomechanical lattice
Source: Light Sci Appl. 2025 Feb 2;14:66. doi: 10.1038/s41377-024-01736-3 (PMC11788426; doi:10.1038/s41377-024-01736-3)
Supplement: Supplementary file 1 — Retiming dynamics of harmonically mode-locked laser solitons in a self-driven optomechanical lattice [file 41377_2024_1736_MOESM1_ESM.docx]

Supplementary Information for
“Retiming dynamics of harmonically mode-locked laser solitons in a self-driven optomechanical lattice”

Xiaocong Wang^1,2,3,†^, Benhai Wang^2,†^, Wenbin He^2,*^, Xintong Zhang^1,2,3^, Qi Huang^2^, Zhiyuan Huang^3^, Xin Jiang^2^, Meng Pang^1,2,3,4**^,Philip. St. J. Russell^2^

^1^Department of Optics and Optical Engineering, University of Science and Technology of China, Hefei 230026, China

^2^Russell Centre for Advanced Lightwave Science, Shanghai Institute of Optics and Fine Mechanics and Hangzhou Institute of Optics and Fine Mechanics, Chinese Academy of Sciences, Shanghai 201800, China

^3^State Key Laboratory of High Field Laser Physics and CAS Center for Excellence in Ultra-intense Laser Science, Shanghai Institute of Optics and Fine Mechanics CAS, Shanghai 201800

^4^Hangzhou Institute for Advanced Study, University of Chinese Academy of Sciences, Hangzhou 310024, China

^*^Corresponding author, emails: wenbin.he@r-cals.com, pangmeng@siom.ac.cn

^†^These authors contribute equally to the work

1. Perturbation dynamics of control pulses

The control pulses we used for perturbing the intra-cavity solitons are generated by modulating a 1550 nm single frequency CW laser (NKT Koheras, linewidth <100 Hz) using an electro-optical modulator, as shown in Fig. 4 in the main text. The duration of externally launched pulses is 70 ps, which is ~50 times wider than soliton, ensuring sufficient overlapping between the control pulses and the perturbed solitons when they co-propagate in the SMF. The control pulses were generated at intervals exactly corresponding to the cavity round-trip time of the mode-locked lasers, in order to perturb the selected soliton repeatedly. The control pulses were amplified to gain a peak power of ∼10 W so as to ensure the strength of XPM effect with the solitons. An optical switch was used to set a 200 μs time window for the perturbation (with a rise/fall time of 300 ns). Two FPCs and an inline polarizer was used to adjust the polarization states of the control pulses in order to eliminate the control pulses at the inline polarizer in the mode-locked cavity and thus avoiding further perturbation in the EDFA section.

The perturbation exerted by the control pulses not only induced deviations in the position and group velocity of the perturbed soliton, but also leads to temporary attenuation of the soliton energy, as already demonstrated in our previous work [1, 2]. Due to the properties of laser solitons [3], the decrease in its energy would cause spectral bandwidth narrowing simultaneously, which would then affect the damping strength of their retiming oscillation in the optomechanical lattice. The spectral bandwidth narrowing of the perturbed solitons can be revealed using the DFT method, which linearly maps the profile of their optical spectrum into the time domain [4]. We used a 5-km-long SMF for dispersive stretching of the output soliton sequence at OC-2. Two examples are provided below, as shown in Fig. S1. In the first example (Fig. S1a), the control pulses overlapped with the solitons with a slight discrepancy in the repetition rate, dragging the soliton away from the balanced position [1], meanwhile causing some decrease in the energy (above the critical energy $E_{c}$) [2]. The DFT signal (shown in Fig. S1b) clearly shows the narrowing of the soliton spectrum after the perturbation, although both the energy and bandwidth recovered later during the retiming of the solitons. In the second example (Fig. S1c), the control pulses exerted stronger perturbation upon the selected soliton, causing significant reduction in the soliton energy (below $E_{c}$). In this case the soliton can no longer recover, while we can see from the DFT signal that the soliton bandwidth kept narrowing as the soliton gradually vanished. Note that Fig. S1a and Fig. S1c are recorded at OC-1 in order to show the relative position of control pulses and the perturbed solitons. Meanwhile the Fig. S1b and Fig. S1d are recorded at OC-2 before which the control pulses have already been eliminated in order to clearly show the DFT signal of the perturbed soliton.


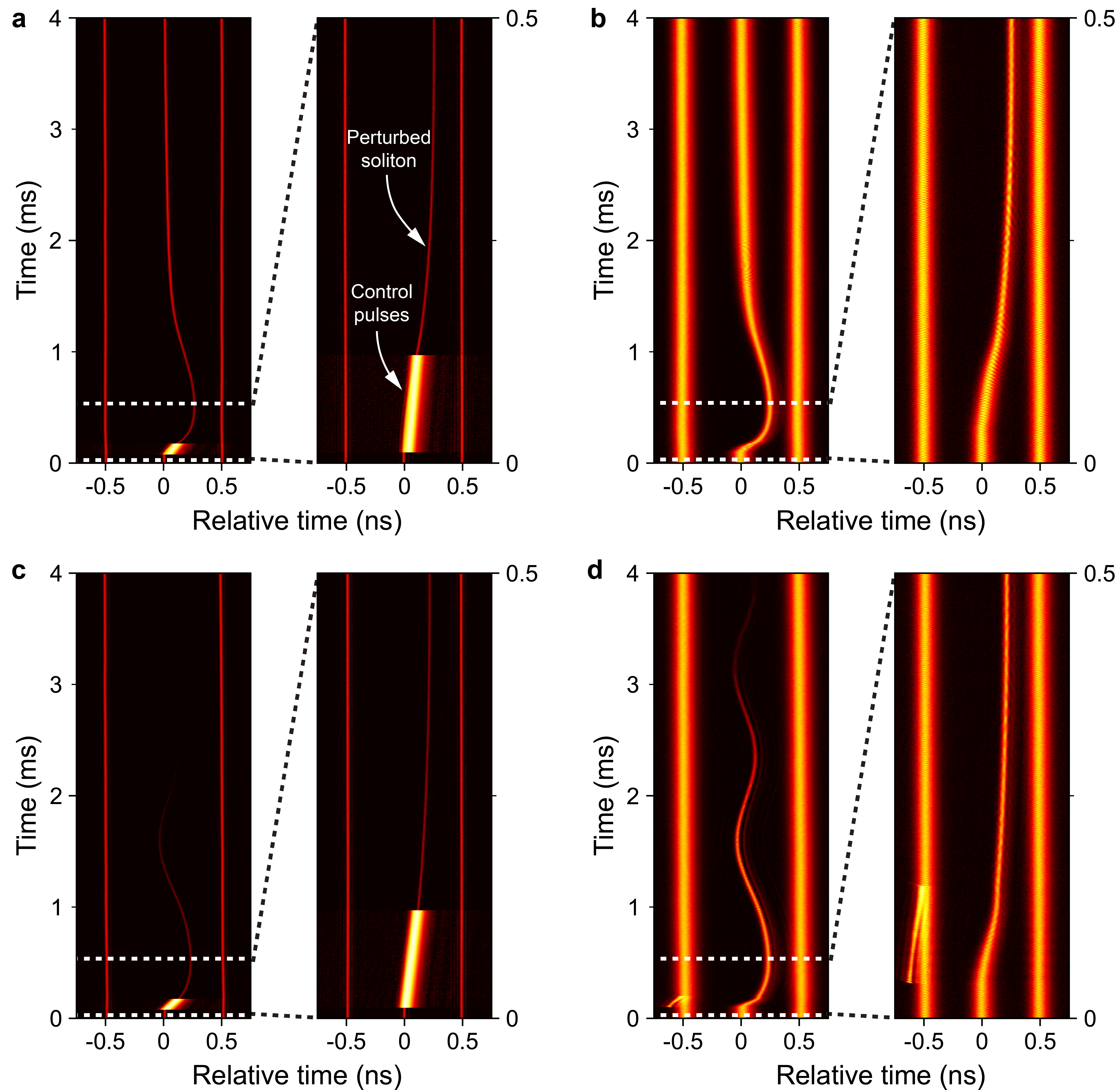


**Fig. S1 |** **a** Perturbation and subsequent over-damped oscillatory retiming of the selected soliton recorded at OC-1. The perturbation region is zoomed-in in the right panel for clear illustration of the relative position between the control pulses and the perturbed soliton. Note that the two next-neighbor solitons remain unperturbed. **b** The DFT signal corresponding to **a** recorded at OC-2. **c** Perturbation by control pulses was increased, which caused vanishing of the perturbed soliton during the oscillatory retiming. **d** The DFT signal corresponding to **c** recorded at OC-2. Note that slight leakage of the control pulses was observed in the DFT signal, while the leakage did not cause obvious perturbations upon the other solitons in the cavity.

1. The period of retiming oscillations

The period of the retiming oscillations of the perturbed soliton is determined by the restoring “force” induced by the trapping potential, which can be varied under different cavity parameters. According to Eq. S12 in Ref. [2], under linear approximation, the restoring force $F\left( \Delta\tau\right)$ given in Eq. (4) in the main text is related to the temporal deviation $\Delta\tau$ according to:

| $F\left( \Delta\tau\right)=-\frac{\omega_{0}{\Delta n}^{''}L_{\mathrm{PCF}}\beta_{2}^{\mathrm{ave}}L_{R}}{c}\Delta\tau$ |  | (S1) |
| --- | --- | --- |

in which $\Delta\tau$ is the temporal deviation of the soliton from the balanced position normalized to the cavity round-trip time $T_{R}$ (i.e. $\Delta\tau= \left( t-t_{0} \right)/{T_{R}}$ where $t_{0}$ is the balanced position), $\omega_{0}$ the carrier frequency, $L_{\mathrm{PCF}}$ the PCF length, $\beta_{2}^{\mathrm{ave}}$ the average cavity GVD (which is a negative value in unit of ps^2^ km^-1^), $L_{R}$ the cavity length, and $c$ the vacuum light speed, $\Delta n^{''}$ is the second-order Taylor coefficient of the index modulation induced by the acoustic vibration in the PCF at the balanced position of the laser soliton. The Taylor expansion of the index modulation in real time scale about the balanced position up to the second order term can be written as below:

| $\Delta n\left( t-t_{0} \right)= {\Delta n}_{0}+{\Delta n}^{'}\left( t-t_{0} \right)+\frac{1}{2}{\Delta n}^{''}({t-t_{0})}^{2}$ |  | (S2) |
| --- | --- | --- |

in which ${\Delta n}_{0}$, ${\Delta n}^{'}$, and ${\Delta n}^{''}$are the Taylor coefficients for the expansion terms from the zeroth to the second order (${\Delta n}^{''}$ has a unit of $\sec^{-2}$). Stable trapping of the soliton in the acoustic lattice requires that ${\Delta n}^{''}<0$. As a result, the repetition rate is locked at a frequency lower than the resonance frequency, typically at the FWHM position of the acoustic resonance [2].

The dimensionless coefficient of $\Delta\tau$ in the right-hand side of Eq. (S1) is simply the squared angular frequency of the retiming oscillation enforced by the trapping potential. We can then roughly estimate the corresponding oscillation period using the following practical numbers. We can calculate that ${\Delta n}^{''}$ is in the order of ${-10}^{-13} \mathrm{ps}^{-2}$ given that a 2 GHz vibration in the PCF-core leads to a ${\Delta n}_{0}$ in the order of ${10}^{-8}$ along the PCF [5]; the total group delay $\beta_{2}^{\mathrm{ave}}L_{R} \approx-0.5 \mathrm{ps}^{2}$ ($\beta_{2}^{\mathrm{ave}}\approx-20$ps^2^ km^-1^ and $L_{R} \approx25$ m), and ${\omega_{0}}/c={2\pi}/{\left( 1.55 \mu m \right)\approx4{\mu m}^{-1}}$. Using the expression of restoring force given in Eq. (S1), we can estimate that the oscillation period is $\sim{10}^{4}$ in normalized time scale $\tau$ with respect to the round-trip time ($T_{R}\approx100 \mathrm{ns}$ for 25-m-length cavity), which is roughly in the order of 1 ms in the real time scale, agreeing well with our experimental observation.

The oscillation period can be varied under different cavity configurations, as indicated by Eq. (S1), which have also been verified in our experiments. Two examples are provided in Fig. S2 which gives the retiming oscillations under two different cavity configurations. In the first example (Fig. S2a), we have $L_{\mathrm{PCF}}=0.7 m$ and $L_{R}=24 m$, and we can observe that the retiming oscillation lasted for four cycles over ~6.5 ms, giving an oscillation period of ~1.6 ms. In the second example (Fig. S2b) we have increased the fiber length such that $L_{\mathrm{PCF}}=1.5 m$ and $L_{R}=35 m$, (thus $\beta_{2}^{\mathrm{ave}}$ remained almost unvaried). We can notice that due increase of the PCF length and the cavity length, the restoring “force” is enhanced and thus oscillation period has been reduced significantly to ~ 1.2 ms (four cycles of oscillations are observed over a time span of ~ 4.6 ms). According the Eq. (S1), if we assume that ${\Delta n}^{''}$ is unvaried, the scaling in the oscillation period should be about 1.21 times (1.77 times in normalized time scale, while $T_{R}$ are different in these two cases by 1.46 times), which agrees quite well with the experimental results that yielded a scaling of 1.33 times (from 1.2 to 1.6 ms). The scaling in oscillation period does not fully match the change in the fiber length probably due to the slight difference of ${\Delta n}^{''}$ in these two cases, which is sensitive to both the magnitude of the acoustic vibration and the frequency detuning of the repetition rate, and is meanwhile difficult to characterize precisely in experiments.


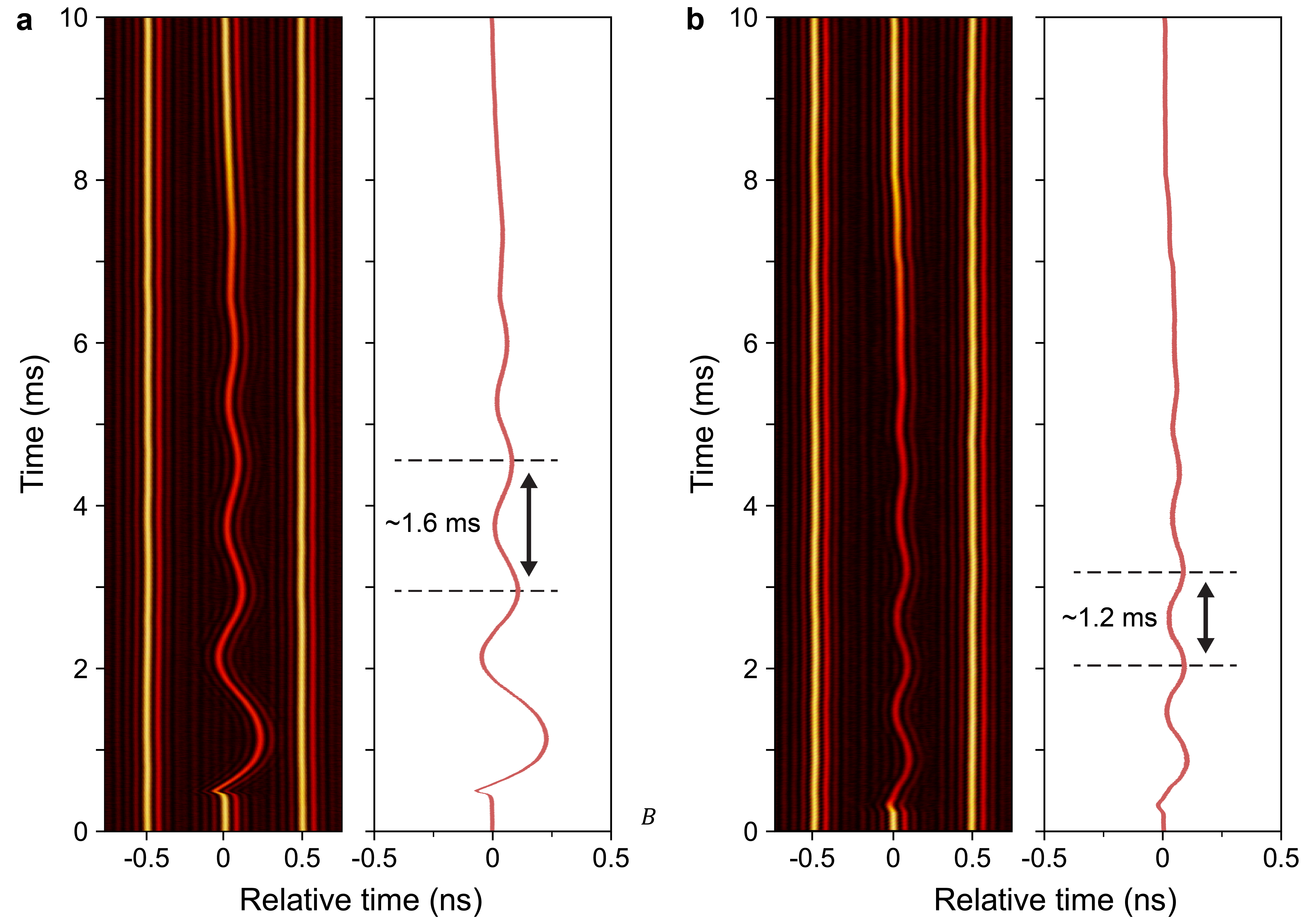


**Fig. S2 |** Retiming oscillations observed in two different cavity configurations. **a** The cavity includes a 0.7-m-long PCF and has a 24-m-long cavity length. The observed retiming oscillation period is ~1.6 ms. The trajectory of the perturbed soliton is extracted and plotted separately on the right panel for clear illustration. **b** The cavity includes a 1.5-m-long PCF and has a 35-m-long cavity length. The observed retiming oscillation period is ~1.2 ms.

In fact, due to the sinusoidal profile of the acoustic lattice, the restoring force induced by the acoustic lattice becomes nonlinearly related to the position deviation $\Delta\tau$ given large initial deviations. The restoring force around the balanced position can then be expressed using Taylor expansion as:

| $F\left( \Delta\tau\right)=k_{1}\Delta\tau+k_{2}{\Delta\tau}^{2}+k_{3}{\Delta\tau}^{3}+\ldots$ |  | (S3) |
| --- | --- | --- |

Due to the nonlinear terms in $F\left( \Delta\tau\right)$, the trajectory of retiming oscillation in the time domain observed in our experiments are slightly different from a damped sinusoidal trajectory. We have revealed from numerical calculations that the profile of the first cycle in the retiming oscillation could be sensitive to the initial deviations (position and velocity) due to the existence of these nonlinear terms. As the oscillation quickly damped to the close vicinity of the balanced position, the trajectory can then be well described using a harmonic potential assumption. Further investigations are needed to gain better insights into the nonlinear properties of the restoring forces induced by the acoustic lattice.

1. Dissipative model of retiming oscillation

We have developed a phase-space description and a corresponding dissipative model (Eqs. (1) – (4)) that describe the retiming oscillation of the laser soliton. The key mechanism in this model is that the presence of a trapping potential leads retiming oscillation of deviated solitons, while the damping strength of the retiming oscillation is determined by the soliton bandwidth through gain filtering effect. The soliton bandwidth is directly proportional to the soliton energy due to the soliton-area theorem [3] which implies $E\propto1/\tau\propto B_{s}$. Meanwhile the dynamics of soliton energy is governed by the balance between the EDFA gain and the NPR-induced loss as described Eq. (1) in the main text. In this way, the retiming dynamics are coupled to the dissipative dynamics of the intra-cavity solitons. We employ the following assumption concerning the dissipative terms in Eq. (1) for the numerical simulations given in Fig. 6c in the main text. Firstly, the EDFA gain $g$ is related to the soliton bandwidth $B_{s}$ (and thus to the soliton energy $E$) through gain filtering effect according to:

| $g\left( B_{s} \right)=g_{0}\left( 1-\frac{{B_{s}}^{2}}{\Omega_{g}^{2}} \right)=g_{0}\left( 1-\frac{\left( k_{s}E \right)^{2}}{\Omega_{g}^{2}} \right)$ |  | (S4) |
| --- | --- | --- |

where we assumed a quadratic dependence of the gain coefficient upon the soliton bandwidth [6] with $g_{0}$ being the gain for quasi-CW pulse and $\Omega_{g}$ is the gain bandwidth, and $k_{s}$ a heuristic constant. This assumption is valid given that the soliton bandwidth is small compared with the gain filtering bandwidth. Note that the gain saturation effect can also lead to lower gain for higher soliton energy, which can further decrease the gain coefficient when the soliton energy is increased.

Secondly, the NPR-induced loss is related to the peak power of the soliton $P_{s}$ (and thus the soliton energy $E$ since $P_{s}\propto E^{2}$ ) sinusoidally, and the complete form of $\alpha\left( P_{s} \right)$ used in numerical simulation is given by [7]:

| $\alpha\left( P_{s} \right)=\alpha\left( k_{\alpha}E^{2} \right)=\alpha_{1}+\alpha_{0}\sin(k_{\alpha}E^{2}+b_{\alpha})$ |  | (S5) |
| --- | --- | --- |

in which $\alpha_{1}$ is the unsaturable loss, $\alpha_{0}$ is a constant related to the magnitude of the NPR-induced loss, $k_{\alpha}$ indicates the sensitivity of the NPR-loss to the soliton energy, $b_{\alpha}$ indicates the polarization bias of the cavity, which needs to be adjusted to form proper values of $E_{s}$ and $E_{c}$ as shown in Fig. 6a in the main text.

At last, the damping strength $\Gamma$ is affected by $B_{s}$ due to the gain filtering effect. This effect can be illustrated using the conceptual sketch in Fig. S3. The initial change of the group velocity $\Delta v_{g}$ of the perturbed soliton can regarded as the consequence of a change in the carrier frequency $\Delta\omega$ of the soliton following $\Delta v_{g}=-\beta_{2}^{\mathrm{ave}}v_{g0}^{2}\Delta\omega$ ($v_{g0}$ is the unperturbed soliton group velocity). The gain filtering effect provided by the EDFA tends to eliminate $\Delta\omega$ by pulling the center of the soliton spectrum back to that of the gain spectrum, as conceptually illustrated in Fig. S3a. During this process, $\Delta v_{g}$ is also decreased, leading to an effective damping for the oscillation motion of the soliton in the trapping potential (See Fig. S3).


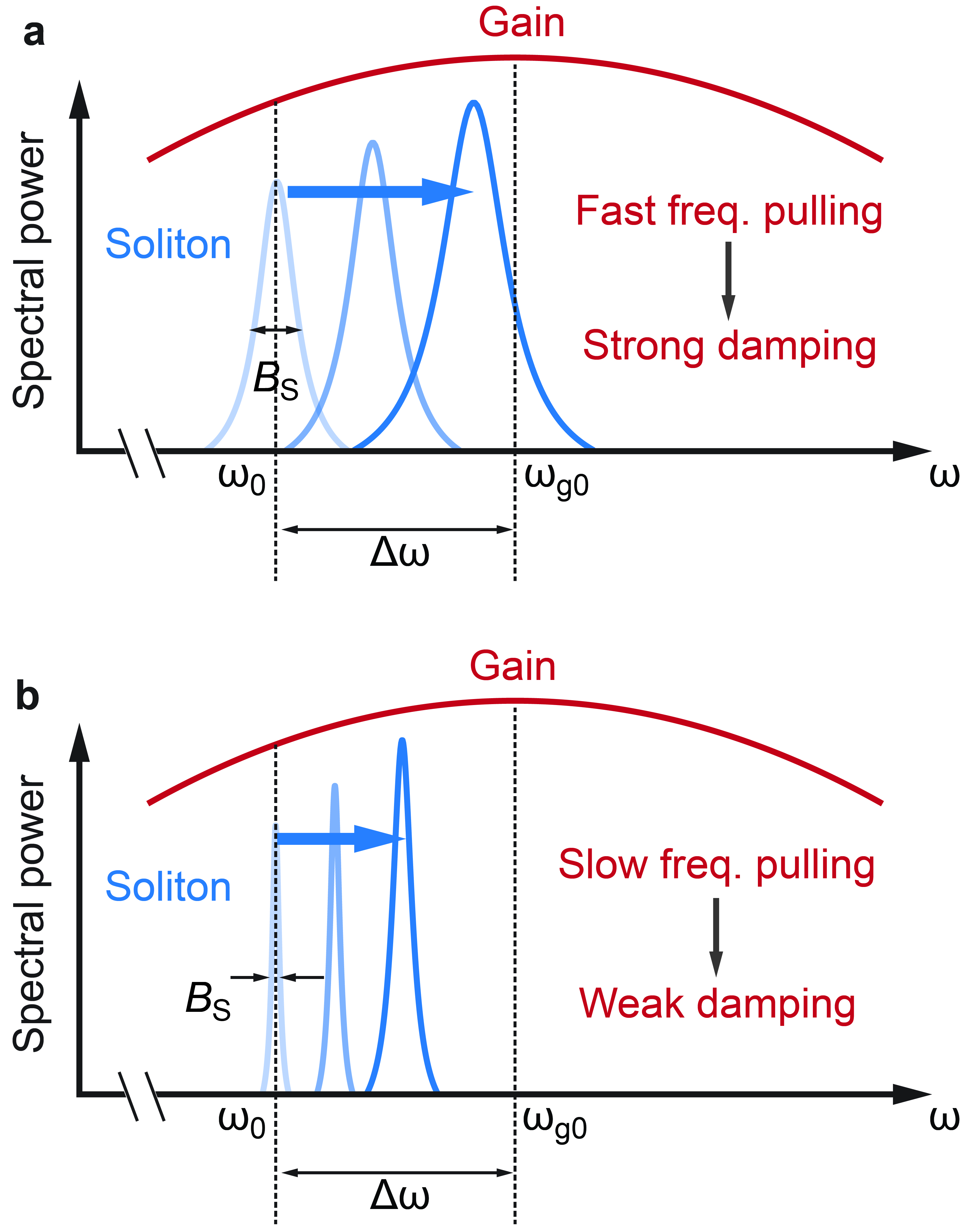


**Fig.S3** | Conceptual illustration of the bandwidth-dependent damping strength for soliton retiming. **a** Soliton spectrum with a bandwidth of $B_{s}$ and a shifted carrier frequency $\omega_{0}$ away from the from the spectral center of the EDFA gain $\omega_{g0}$ by $\Delta\omega$. The gain filtering effect tends to pull the center of the soliton spectrum back to the center of the gain by eliminating $\Delta\omega$, which causes the damping of the soliton motion. **b** Given a narrower soliton bandwidth, the pulling of the soliton spectrum is much slower with each amplification per round-trip, leading to weaker damping compared to **a**. Note during the amplification the soliton energy gradually increase, leading to broader bandwidth.

As a consequence, we can expect that solitons with larger bandwidth would be more sensitive to the unbalanced gain profile away from the spectral center of the gain, and thus would be damped more strongly during the retiming oscillation. As a simple approximation, we assume the damping strength depends quadratically upon $B_{s}$ (and thus $E$) as [8]:

| $\Gamma\left( B_{s} \right)=k_{\Gamma}E^{2}$ | , | (S6) |
| --- | --- | --- |

As a results, a much lower damping strength would be expected if the soliton energy is reduced significantly during the perturbation due to the correspondingly narrowed soliton bandwidth, as illustrated in Fig. S3b. For the case with a vanishing soliton, the damping strength could become negligible, and the soliton would keep oscillating even when it has almost disappeared in the noise background of the laser field. Nevertheless, comprehensive understanding of the damping mechanism would require further investigation, while the dissipative model provided in this work can serve as a simple guide to understand the dominant factors in the retiming dynamics of the solitons in the acoustic lattice.

References

1. He, W. B. et al. Synthesis and dissociation of soliton molecules in parallel optical-soliton reactors. *Light*: *Science & Applications* **10**, 120 (2021).

2. Pang, M. et al. All-optical bit storage in a fibre laser by optomechanically bound states of solitons. *Nature Photonics* **10**, 454-458 (2016).

3. Pang, M. et al. Stable subpicosecond soliton fiber laser passively mode-locked by gigahertz acoustic resonance in photonic crystal fiber core. *Optica* **2**, 339-342 (2015).

4. Goda, K. & Jalali, B. Dispersive Fourier transformation for fast continuous single-shot measurements. *Nature Photonics* **7**, 102-112 (2013).

5. Kang, M. S. et al. Tightly trapped acoustic phonons in photonic crystal fibres as highly nonlinear artificial Raman oscillators. *Nature Physics* **5**, 276-280 (2009).

6. Haus, H. A. Mode-locking of lasers. *IEEE Journal of Selected Topics in Quantum Electronics* **6**, 1173-1185 (2000).

7. Ippen, E. P. Principles of passive mode locking. *Applied Physics B* **58**, 159-170 (1994).

8. Grein, M. E. et al. Timing restoration dynamics in an actively mode-locked fiber ring laser. *Optics Letters* **24**, 1687-1689 (1999).
